# Effectiveness of Pain Neuroscience Education in Physical Therapy: A Systematic Review and Meta-Analysis

**DOI:** 10.3390/brainsci15060658

**Published:** 2025-06-18

**Authors:** Andrea Sánchez-Robalino, Hugo Sinchi-Sinchi, Andrés Ramírez

**Affiliations:** 1 Department of Physiotherapy, Pontificia Universidad Católica del Ecuador Sede Esmeraldas, Esmeraldas 080101, Ecuador; asanchez898@pucese.edu.ec; 2 Department of Psychology, Pontificia Universidad Católica del Ecuador Sede Esmeraldas, Esmeraldas 080101, Ecuador; hfsinchi@pucese.edu.ec; 3 Department of Clinical Psychology, Grupo de Investigación en Neurociencia Clínica Aplicada (GINCA), Universidad Politécnica Salesiana, Cuenca 010107, Ecuador

**Keywords:** pain neuroscience education, physiotherapy, chronic pain, systematic review, meta-analysis

## Abstract

**Background**: Pain neuroscience education (PNE), when combined with physical therapy interventions, has been recognized as an effective strategy for improving pain management and reducing disability in individuals with chronic pain. **Objective**: This systematic review and meta-analysis aimed to evaluate the effectiveness of PNE in combination with rehabilitation modalities, with a focus on pain reduction and functional improvement. **Methods**: A comprehensive systematic search was conducted in Cochrane, PsycInfo, PubMed, ScienceDirect, Scopus, and Web of Science databases to identify randomized clinical trials examining the effects of combining PNE with physical therapy. Nineteen studies met the inclusion criteria. Data extraction focused on demographic and methodological characteristics, as well as outcomes related to pain and disability. **Results**: The findings indicate that PNE combined with physical therapy significantly reduces pain intensity and enhances functionality. The mean pain score decreased from 5.89 (pre-intervention) to 3.03 (post-intervention), with similar improvements observed in disability outcomes. However, heterogeneity among studies—attributable to sociocultural and methodological differences—suggests the need for a cautious interpretation of the results. **Conclusions**: The integration of PNE with physical therapy appears to be an effective approach for reducing pain and improving functional outcomes in patients with chronic pain. Nevertheless, further research is recommended to address existing heterogeneity and to refine standardized intervention protocols.

## 1. Introduction

The World Health Organization [1] reported that approximately 1.17 billion people worldwide suffer from chronic pain. In Ecuador, there are an estimated 5000 prevalent cases of chronic pain per 100,000 inhabitants. This type of pain often persists, limiting functionality, the ability to work, and social participation [1]. In 2020, the International Association for the Study of Pain updated its definition of pain, describing it as “an unpleasant sensory and emotional experience associated with, or resembling that associated with, actual or potential tissue damage”. This revision introduced important clarifications, emphasizing that pain is a subjective and individual experience, which may not necessarily correspond to observable tissue damage [2]. It is also noteworthy that morphological changes occur at the cortical level of the central nervous system, affecting structures critical for human movement and emotional regulation [3].

Recent studies highlight the importance of various physical therapy modalities for managing chronic pain. Their application has demonstrated value in promoting, maintaining, and restoring the health and well-being of patients with chronic pain across multiple dimensions [4]. One emerging strategy aimed at improving patients’ quality of life, alongside physical therapy, is pain neuroscience education (PNE). According to the Medical Subject Headings (MeSH), cognitive neuroscience—the broader field encompassing PNE—studies mental processes related to learning and the corresponding brain regions involved. This educational approach has been referred to by several synonymous terms, including pain education, Neurophysiological Pain Education, Cognitive Behavioral Therapy, and pain neuroscience education [5]. Its goal is to increase patients’ understanding of pain and the nervous system, ultimately helping them reconceptualize their pain experience [6].

Several systematic reviews support combining PNE with physical therapy. For instance, Wood et al. [4] provide moderate evidence for its use in patients with chronic low back pain, Bonatesta et al. [7] support its application in patients with non-specific chronic spinal pain, and Goff et al. [8] conclude that patient education should not be offered in isolation but rather combined with therapeutic exercise. However, these studies are often limited by a small number of trials, leaving notable gaps in the literature. A major challenge that remains is the adaptation of this educational approach to different patient populations, considering the biopsychosocial complexities that influence pain perception, which further reinforces the need for research from a holistic perspective [9,10].

This study aims to contribute to the understanding of the effectiveness of pain neuroscience education (PNE) in physical therapy clinical practice and its impact on treatment outcomes. The objective of this systematic review and meta-analysis was to evaluate the scientific evidence regarding the effectiveness of PNE combined with clinical rehabilitation modalities, focusing on its impact on pain management and functional outcomes. The specific objectives were as follows: (a) to determine the efficacy of these interventions in reducing pain intensity and disability and (b) to identify the most used instruments for measuring pain intensity and disability. Chronic pain is the condition most frequently managed by physiotherapists, irrespective of the patient’s underlying pathology or affected region. This review introduces pain neuroscience education (PNE) as an adjunct to conventional physiotherapy and advocates for its broader integration into clinical practice.

## 2. Methods

This systematic review and meta-analysis was conducted in accordance with the guidelines of the PRISMA 2020 statement [11,12].

Population: Studies involving participants aged 18 years or older with chronic pain. Intervention: Pain Neuroscience Education (PNE) combined with a physiotherapeutic modality. Comparison: Studies comparing patients who received a physiotherapeutic intervention such as physical agents, manual therapy, therapeutic exercise, or physical activity. Outcome: Measurements of pain and disability. Study Design: Randomized clinical trials reporting pre- and post-test measures of pain.

*Inclusion criteria:* (1) Participants aged 18 years or older; (2) studies employing pain neuroscience education (PNE); (3) studies comparing intervention modalities; (4) studies reporting quantitative outcomes; (5) studies using validated instruments to measure pain and disability; (6) studies reporting pre- and post-test means; (7) randomized clinical trials.

*Exclusion criteria:* (1) Studies involving participants who did not meet the age requirement; (2) clinical trials without PNE combined with a physical therapy modality; (3) studies lacking comparison with any physiotherapeutic intervention; (4) studies not reporting measurements of pain or disability; (5) studies of the literature review type; (6) studies without pre- and post-test measurements.

*Search strategies*

The search was conducted in March 2024 across six electronic databases, Cochrane, PsycInfo, PubMed, ScienceDirect, Scopus, and Web of Science, chosen for their relevance and international scope in health sciences research. The search strategy was developed by combining terms derived from the PICOS framework with the most frequently cited keywords in the literature, including the following: “Chronic Pain”, “Musculoskeletal Pain”, “Chronic Disease”, “Physical Therapy Modalities”, “Pain Management”, “Rehabilitation”, “Education”, “Neurosciences”, and “Neurobiology.” Boolean operators “AND” and “OR” were used to optimize the search and maximize the retrieval of relevant studies (see Supplementary S1).

*Selection of studies*

Search results were independently reviewed by two investigators (AS and HS). After ensuring congruence, records were exported in BibTeX (for PubMed) and RIS (for Scopus, Web of Science, and PsycInfo) formats. Records were coded using the following labels: duplicate, deleted, included, and maybe. Specific reasons for inclusion or exclusion were documented. The entire screening and selection process was managed using the Rayyan web platform [11].

*Data extraction*

Data extraction focused on two categories: (1) sociodemographic characteristics of the studies and (2) methodological characteristics. The researchers defined the extraction variables in advance and cross-checked the information to resolve any inconsistencies.

Microsoft Excel was used to develop the working matrix, which facilitated the organization of key information from potentially relevant articles. Extracted variables included the following: author, year of publication, title, journal, quartile ranking (SJR), country, continent, study design, population (type of pain), sample size (intervention and control groups), sex distribution (intervention and control groups), mean age (intervention and control groups), type of intervention (intervention and control groups), duration of intervention (intervention and control groups), pain measurement instrument (intervention and control groups), disability measurement instrument (intervention and control groups), mean and standard deviation of pain scores (pre-intervention and post-intervention), and mean and standard deviation of disability scores (pre- and post-intervention).

Pain was evaluated primarily with two well-validated instruments: the Numerical Pain Rating Scale (NPRS) and the Visual Analog Scale (VAS). The NPRS asks participants to rate their pain on an 11-point scale from 0 (“no pain”) to 10 (“worst pain imaginable”). The VAS consists of a 10 cm horizontal line anchored by the same descriptors at each end; respondents mark a point along the line corresponding to their pain intensity. When studies reported pain on a 0–100 scale, we converted scores proportionally to the 0–10 metric to enable direct comparisons and to pool results in the meta-analysis. This harmonization ensures that all pain outcomes reflect the same underlying construct, facilitating more accurate cross-study synthesis and interpretation.

*Risk of Bias Evaluation*

To ensure the methodological quality of the meta-analysis, only randomized clinical trials (RCTs) that used pre- and post-intervention assessments and validated instruments for measuring pain and disability were included. Furthermore, only studies reporting both mean and standard deviation values for pain and disability were considered, allowing for standardized comparisons across studies. Risk of bias in the RCTs was assessed using the PEDro scale, which evaluates internal and external validity across 11 criteria. Higher PEDro scores correspond to greater methodological quality. Two investigators (AS-R and HS-S) independently assessed the methodological quality of the 19 included studies. In cases of disagreement, a third investigator (AR) served as a referee to resolve discrepancies, ensuring additional quality control and minimizing potential biases in the selection and analysis processes (see Supplementary S2).

*Certainty of evidence assessment*

The certainty of the evidence was evaluated using the GRADE (Grading of Recommendations, Assessment, Development, and Evaluation) approach. The five GRADE domains—risk of bias, inconsistency, indirectness, imprecision, and other considerations—were assessed for each outcome. Based on this evaluation, the overall certainty of evidence was categorized as high, moderate, low, or very low. The Summary of Findings table was created using GRADEpro GDT software (see Supplementary S3).

*Statistical Analysis*

The statistical analysis was based on the standardized mean difference (SMD) as the primary outcome measure. A random effects model was fitted to account for variability across studies. The degree of heterogeneity was estimated using the restricted maximum likelihood (REML) method [13]. Additionally, Cochrane’s *Q* statistic [14] and the I^2^ index were calculated to assess heterogeneity. When any degree of heterogeneity was detected (i.e., tau^2^ > 0), a prediction interval for the true effects was provided.

Statistical tests and confidence intervals were computed using the Knapp and Hartung method. Residual analysis and Cook’s distance were employed to detect outliers and influential studies. Studies with residuals exceeding the Bonferroni-corrected critical value (i.e., 100 × [1 − 0.05/(2 × k)] percentile of a standard normal distribution) were considered potential outliers. Studies with a Cook’s distance greater than the median plus six times the interquartile range were classified as influential. Funnel plot asymmetry was assessed using both a rank correlation test and a regression test, with the standard error of the observed outcomes as a predictor. All statistical analyses were performed using *Jamovi* software version 2.4.8 and R statistical software version 2023.09.1+494 [15].

The methodological decisions in this review—such as the use of the random effects model, the inclusion of only RCTs with validated instruments, and the application of the PEDro scale—were made to enhance the internal validity and comparability of the findings. Additionally, the use of the PICOS framework and standardized effect size (SMD) is supported by prior reviews on chronic pain, facilitating synthesis across heterogeneous interventions.

## 3. Results

Of the 2431 documents initially retrieved in Rayyan, 988 duplicate records (more than 90% content overlap) were removed using algorithmic and manual detection methods. Following a rigorous title screening of 1443 articles based on the inclusion and exclusion criteria, 1250 articles were excluded for reasons such as being literature reviews, involving different populations, falling outside the age criteria, or being qualitative studies or non-randomized trials.

**Figure 1:**
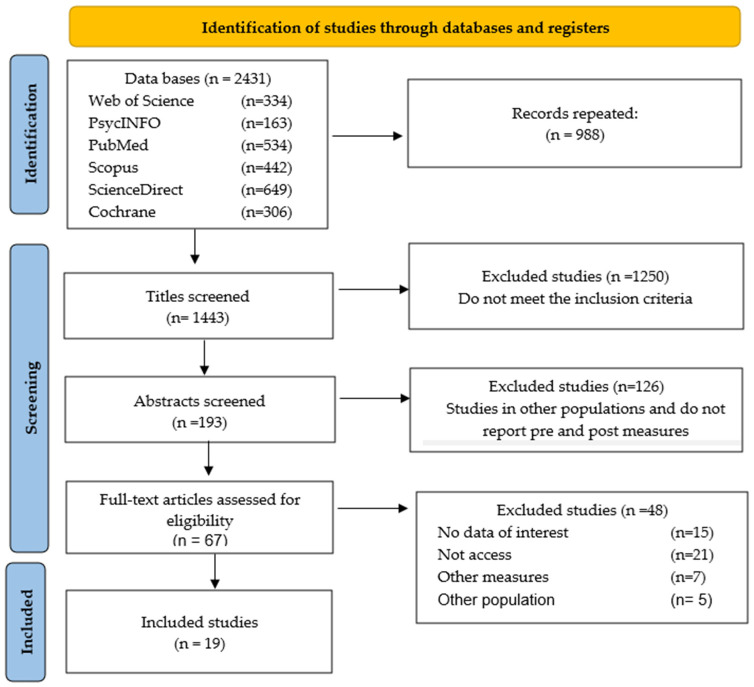
PRISMA flow diagram.

Subsequently, abstracts of 193 articles were assessed in detail, resulting in the exclusion of 126 additional studies for reasons including different populations, a lack of outcome measurements, or the absence of pre- and post-intervention data. Full-text evaluation was then performed for 67 articles by both investigators (AS and HS-S). Any disagreements regarding eligibility were resolved through discussion until consensus was reached. Ultimately, 48 articles were excluded due to reasons such as missing relevant data, an inaccessible full text, population differences, or unsuitable outcome measures (Figure 1).

A total of 19 articles were included in the analysis (Table 1). The highest number of publications occurred in 2023, accounting for seven studies (36.7%). Europe contributed the largest share of studies (seven studies, 36.8%), followed by Asia (six studies, 31.6%). Spain and Iran were the countries with the highest frequency of publications, each contributing three studies (15.8%). Overall, 19 journals were represented, of which 11 (57.9%) were ranked in Quartile 1 (Q1) according to the Scimago Journal Rank (SJR).

### 3.1. Descriptive and Sociodemographic Characteristics of the Studies

The total sample size across all included studies was 693 patients who reported manifestations of chronic pain. The sample sizes across individual studies varied considerably, ranging from 7 to 128 participants, with a mean of 36.47 participants per study (SD = 34.97). The mean age of the participants across the studies was 44.97 years (SD = 8.235), with the individual study mean ages ranging from 20.7 to 58.34 years.

Chronic low back pain was the most frequently investigated condition, reported in eight studies (42.1%) [16,19,20,24,29,30,31,33]. Other pain conditions examined included chronic painful temporomandibular disorders (TMDs) in one study [17], neck pain in two studies [18,26], distal radius fractures in one study [21], chronic spinal pain in one study [22], cervicogenic headache in one study [23], musculoskeletal pain in one study [25], migraine with or without neck pain in one study [27], carpal tunnel syndrome awaiting surgery in one study [28], knee osteoarthritis in one study [32], and neck pain in one study [34]. Most participants across the included studies were reported as female (data on the exact proportion of females per study would further enhance this description).

The duration of the interventions implemented in the studies ranged from 2 to 24 weeks, with a mean intervention duration of 7.21 weeks (SD = 4.86). The number of pain education sessions delivered varied between studies, from 1 to 12 sessions, with a mean of 3.68 sessions (SD = 2.71). All studies included two primary assessment points: one at baseline (pre-intervention) and one following the intervention (post-intervention). Pain intensity was most measured using the Visual Analog Scale (VAS) in 10 studies (52.6%) and the Numerical Pain Rating Scale (NPRS) in 9 studies (47.4%).

Disability outcomes were assessed in 10 studies. The Roland Morris Disability Questionnaire (RMDQ) was utilized in five studies [19,20,22,24,30,31]; the Neck Disability Index (NDI) in two studies [18,23]; the Disabilities of the Arm, Shoulder, and Hand (DASH) in one study [21]; the Chronic Pain Disability Inventory (CPDI) in one study [17]; the Neck Pain and Disability Scale (NPDS) in two studies [26,27]; and the Quebec Back Pain Disability Scale (QBPDS) in one study [29]. One study used the Boston Carpal Tunnel Questionnaire (BCTQ) [28], and one study did not report a disability outcome [25]. A final total of 19 studies were included in this systematic review and meta-analysis.

Pain neuroscience education (PNE), when combined with rehabilitation modalities, has been shown to be effective in significantly improving patient health outcomes, particularly when integrated with therapeutic exercise as the most used modality (52.6% of cases). The data demonstrate a clear improvement in both the pain intensity and disability levels among participants, highlighting the positive impact of these interventions.

Regarding pain outcomes, measurements taken before (pain 1) and after (pain 2) the intervention revealed a notable reduction in pain intensity. The initial mean pain score was 5.89 (SD = 1.117), which decreased to 3.03 (SD = 1.112) post-intervention. This substantial reduction provides strong evidence of the efficacy of PNE interventions. Furthermore, the Shapiro–Wilk normality test confirmed that the pain data were normally distributed at both assessment points, supporting the statistical validity of the findings (Table 2).

In terms of disability outcomes, participants exhibited significant improvement as well. Prior to the intervention, the mean disability score was 56.67 (SD = 158.607), indicating a considerable degree of functional limitation. Following the intervention, the mean disability score decreased to 12.51 (SD = 8.794), reflecting a substantial enhancement in functional capacity. However, it is important to note the considerable variability in the baseline disability scores, suggesting that some included studies reported highly diverse levels of initial disability.

In addition to its effects on pain and disability, pain neuroscience education (PNE) interventions also contributed to improvements in other important aspects of patient health, including reductions in kinesiophobia, pain-related beliefs, and anxiety associated with the pain experience. These findings highlight that, beyond merely reducing pain intensity, PNE has a positive impact on patients’ beliefs and attitudes toward their condition, which may enhance treatment adherence and improve long-term prognosis.

### 3.2. Random Effects Model and Heterogeneity Statistics

Drawing on 19 studies, our meta-analysis produced four models with effect-size estimates of 0.0588 for the first, −0.784 for the second, 1.75 for the third, and −0.435 for the fourth. While the first, second, and fourth models exhibited low standard errors, the third model’s much larger standard error reflects significantly greater variability in its estimates.

Regarding heterogeneity, assessed using Cochrane’s Q statistic and the I^2^ index, Model 1 showed moderate heterogeneity (I^2^ = 69.28%; Q = 56.745; *p* < 0.001). Model 2 exhibited high heterogeneity (I^2^ = 88.58%; Q = 144.398; *p* < 0.001), while Model 3 demonstrated extremely high heterogeneity (I^2^ = 99.91%; Q = 230.573; *p* < 0.001), indicating substantial inconsistency among study results. Model 4 also presented considerable heterogeneity (I^2^ = 85.69%; Q = 99.719; *p* < 0.001).

Models 2 and 4 were identified as the most robust, with fail-safe N values of 1,356,000 and 419,000, respectively, indicating a low probability of publication bias (Table 3). In contrast, Model 1 appeared more susceptible to bias, with a fail-safe N of 0.140. Begg’s test indicated no evidence of publication bias in any of the models, except for a possible indication in Model 2 (*p* = 0.068). Egger’s regression test further confirmed that Models 2 and 4 showed no evidence of publication bias, whereas Model 3 demonstrated a significant level of bias (*p* < 0.001).

A total of k = 19 studies were included in the analysis. The observed standardized mean deviations ranged from −0.9582 to 0.6498, with the majority of estimates (58%) being positive. The estimated standardized mean difference (SMD) based on the random effects model was 0.0588 (95% CI: −0.1303 to 0.2479). Therefore, the mean result did not differ significantly from zero (t(18) = 0.6536, *p* = 0.5217). According to Cochrane’s Q statistic, the true effects appear to be heterogeneous ((Q18) = 56.7448, *p* < 0.0001; tau^2^ = 0.0984; I^2^ = 69.28%). A 95% prediction interval for the true effects ranged from −0.6270 to 0.7446. Thus, although the mean effect was estimated to be slightly positive, in some studies the actual effect could be negative.

An examination of residuals revealed that one study, Núñez et al. [28], had a residual greater than ±3.0078, suggesting it may be a potential outlier within the model. Cook’s distance analysis also identified the study by Núñez et al. [28] as potentially overly influential. However, neither the rank correlation test nor the regression test indicated significant asymmetry in the funnel plot (*p* = 0.6787 and *p* = 0.9005, respectively) (Table 4).

**Figure 2:**
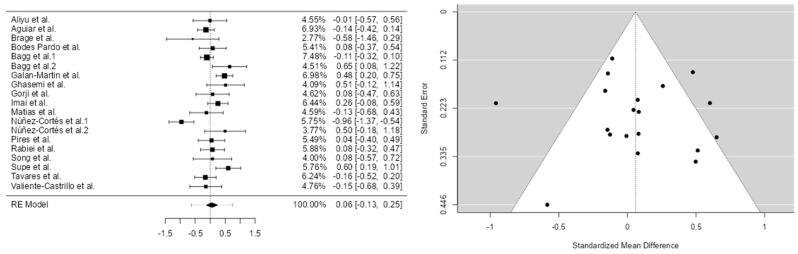
Model 1. Forest diagram and Egger funnel pain 1 [16,17,18,19,20,22,23,24,25,26,28,29,30,31,32,33,34].

The analysis was conducted using the standardized mean difference (SMD) as the primary outcome measure. A random effects model was fitted to the data. The degree of heterogeneity (i.e., tau^2^) was estimated using the restricted maximum likelihood (REML) method [13]. In addition to the estimation of tau^2^, Cochrane’s Q statistic for heterogeneity [14] and the I^2^ index were also calculated [Figure 2].

When any degree of heterogeneity was detected (i.e., tau^2^ > 0), a prediction interval for the true effects was provided. *T*-tests and confidence intervals were computed using the Knapp and Hartung method. Residuals and Cook’s distances were examined to identify potential outliers and/or influential studies within the model.

Studies with a standardized residual greater than the 100 × (1 − 0.05/(2 × k)) percentile of a standard normal distribution were considered potential outliers (applying a Bonferroni correction with a two-sided alpha of 0.05 for the number of included studies, k). Studies with a Cook’s distance greater than the median plus six times the interquartile range (IQR) of Cook’s distances were classified as influential.

The rank correlation test and regression test, using the standard error of the observed outcomes as a predictor, were applied to assess potential funnel plot asymmetry.

The analysis was conducted using the standardized mean difference (SMD) as the outcome measure. A random effects model was applied to the data. The degree of heterogeneity (tau^2^) was estimated using the restricted maximum likelihood (REML) method [13]. In addition to tau^2^, Cochrane’s Q statistic for heterogeneity [14] and the I^2^ index were reported. When heterogeneity was present (tau^2^ > 0), a prediction interval for the true effect size was also calculated. *T*-tests and confidence intervals were computed using the Knapp and Hartung adjustment. Residuals and Cook’s distances were examined to identify potential outliers or overly influential studies.

Studies with standardized residuals exceeding the 100 × (1 − 0.05/(2 × k)) percentile of the standard normal distribution were considered potential outliers (Bonferroni correction, two-sided alpha = 0.05). Studies with Cook’s distance values greater than the median plus six times the interquartile range (IQR) were considered influential. Funnel plot asymmetry was assessed using both the rank correlation test and the regression test, with the standard error of the observed effects as the predictor.

A total of k = 17 studies were included in the analysis. The observed SMDs ranged from −1.3422 to 35.7371, with most estimates being negative (59%). The pooled effect size, calculated using the random effects model, was 1.7522 (95% CI: −2.3457 to 5.8502). This result was not statistically significant (t(16) = 0.9064, *p* = 0.3782).

Cochrane’s Q test indicated substantial heterogeneity (Q(16) = 230.5734, *p* < 0.0001; tau^2^ = 52.6641; I^2^ = 99.91%). The 95% prediction interval ranged from −14.1684 to 17.6728, suggesting that although the overall estimated effect was positive, the true effect may vary considerably across studies and may even be negative in some cases.

**Figure 3:**
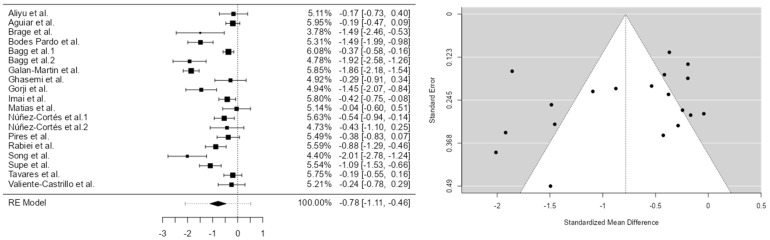
Model 2 Forest diagram and Egger funnel pain 2 [16,17,18,19,20,22,23,24,25,26,28,29,30,31,32,33,34].

An analysis of residuals identified one study, Bagg et al. [20], as a potential outlier (residual > ±2.9738). Based on Cook’s distance, this same study was also considered potentially overly influential. The regression test revealed evidence of funnel plot asymmetry (*p* < 0.0001), while the rank correlation test did not (*p* = 0.2049) [Figure 3].

The analysis was conducted using the standardized mean difference (SMD) as the outcome measure. A random effects model [35] was applied to the data. The degree of heterogeneity (tau^2^) was estimated using the restricted maximum likelihood (REML) method [13]. In addition to tau^2^, Cochrane’s Q statistic for heterogeneity [14,36] and the I^2^ index were reported. When heterogeneity was detected (i.e., tau^2^ > 0), a 95% prediction interval for the true effect sizes was also calculated. *T*-tests and confidence intervals were computed using the Knapp and Hartung adjustment. Residuals and Cook’s distances were examined to identify potential outliers and influential studies.

**Figure 4:**
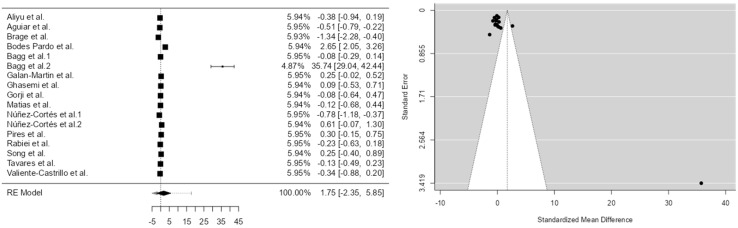
Model 3 Forest diagram and Egger funnel disability 1 [16,17,18,19,20,22,23,24,26,28,29,30,31,33,34].

Studies with standardized residuals exceeding the 100 × (1 − 0.05/(2 × k)) percentile of the standard normal distribution were considered potential outliers (Bonferroni correction, two-sided alpha = 0.05). Studies with Cook’s distance values greater than the median plus six times the interquartile range (IQR) were classified as influential. Funnel plot asymmetry was assessed using both the rank correlation test and the regression test, with the standard error of observed outcomes as the predictor [Figure 4].

A total of k = 17 studies were included in this model. The observed SMDs ranged from −1.1160 to 1.3554, with most estimates (82%) being negative. The pooled standardized mean difference calculated using the random effects model was −0.4352 (95% CI: −0.7362 to −0.1341), indicating a statistically significant difference from zero (t(16) = −3.0642, *p* = 0.0074). Cochrane’s Q test indicated substantial heterogeneity among studies (Q(16) = 99.7193, *p* < 0.0001; tau^2^ = 0.2821; I^2^ = 85.69%). The 95% prediction interval ranged from −1.6006 to 0.7303, suggesting that while the average effect was negative, some studies may report positive effects.

**Figure 5:**
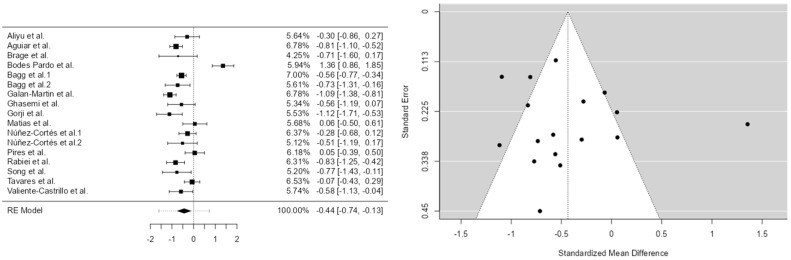
Model 4 Forest diagram and Egger funnel disability 2 [16,17,18,19,20,22,23,24,26,28,29,30,31,33,34].

Residual analysis identified one study [19] with a value greater than ±2.9738, suggesting it may be a potential outlier. According to Cook’s distance, this same study could be considered overly influential in the model. However, neither the rank correlation test (*p* = 0.9032) nor the regression test (*p* = 0.9672) indicated evidence of funnel plot asymmetry [Figure 5].

### 3.3. Sensitivity Analysis and Risk of Bias Assessment

All 19 included studies scored above nine points on the PEDro scale, indicating high methodological quality. Given the absence of low-quality studies, a sensitivity analysis based on risk of bias was not necessary. This uniformity in quality across the included trials strengthens the internal validity and reliability of the pooled estimates derived from the random effects meta-analysis.

### 3.4. Certainty of the Evidence (GRADE Assessment)

The certainty of evidence regarding pain reduction was rated as moderate, based on 19 randomized controlled trials (RCTs). The pooled mean difference (MD) was 0.784 units in favor of pain neuroscience education (PNE) + physiotherapy (95% CI: 0.462 to 1.105), measured on a standardized 0–10 scale using the NPRS or VAS. Despite the precision and consistency of the measurement instruments, the inconsistency across studies (I^2^ = 88.58%) led to downgrading by one level. No concerns were identified regarding the risk of bias, indirectness, or imprecision. The outcome was rated as critical for clinical decision-making, supporting the relevance of this combined intervention.

## 4. Discussion

The objective of this systematic review and meta-analysis was to assess the available evidence on the effectiveness of pain neuroscience education (PNE) combined with clinical rehabilitation modalities and to evaluate its impact on reducing pain and improving functionality in patients with chronic pain.

*(a)*  *Efficacy in the reduction in pain and disability*

There was a decrease in pain intensity, with the mean score dropping from 5.89 pre-intervention to 3.03 post-intervention. These results support the efficacy of the combined approach, aligning with findings from Siddall et al. [37], who observed positive outcomes when physical therapy was paired with exercise, and from Long et al. [38], who highlighted that the duration of effect is related to the length of exposure to the intervention.

Hochheim et al. [39] also found that biopsychosocial interventions produce more favorable outcomes in both pain and disability than purely physical treatments. Likewise, our review demonstrated a striking decrease in disability scores, from a mean of 56.67 at baseline to 12.51 post-intervention. Beyond physical improvements, these interventions enhanced key psychological variables—such as kinesiophobia and pain-related beliefs—which are essential for promoting treatment adherence and sustaining long-term benefits. For example, Salazar et al. [40] showed that a 100 min pain neuroscience education session effectively reduced kinesiophobia. Similarly, Bagg et al. [20] reported that staged treatment protocols yielded positive effects on pain and functional capacity. In contrast, although Pires [29], Aguiar [17], and Ghasemi [23] documented reductions in pain perception, functional gains were less evident in the short term, though some studies did observe longer-term benefits.

The heterogeneity observed in this review may be attributed to variations in sample sizes, intervention types, and geographic contexts, as noted by Deeks et al. [41]. Linden [42] and Ilmawan also emphasized that although PNE was a common component across studies, the results varied considerably, likely reflecting these contextual differences.

In our model analysis, moderate heterogeneity was observed in Model 1 and substantial heterogeneity in Model 3 [43]. This variability may be due to the inclusion of studies with cultural, social, demographic, and methodological diversity. As a result, the findings should be interpreted with caution. Future studies are encouraged to reduce this variability by better matching sociocultural and methodological factors, thereby improving the consistency and comparability of results [44].

Moreover, the risk of bias assessment using the *PEDro scale* revealed that all included studies scored above nine, reflecting high methodological quality across the evidence base. Given this homogeneity, a sensitivity analysis based on methodological quality was not required. This consistency reinforces the robustness of the meta-analytic findings and minimizes concerns regarding internal validity.

Based on the GRADE assessment, the overall certainty of evidence for pain reduction was considered moderate, primarily due to substantial heterogeneity among studies. Nevertheless, the statistically significant mean difference and the robustness of the measurement instruments strengthen the confidence in the observed effects. This supports the clinical utility of combining pain neuroscience education with physiotherapeutic modalities for chronic pain management.

*(b)*  *Instruments used to measure pain and disability*

Across the selected studies, pain perception was measured using either the Visual Analog Scale (VAS) in 10 studies or the Numerical Pain Rating Scale (NPRS) in 9 studies. The choice of instrument was based on ease of use and applicability in clinical practice, underscoring their practicality for pain assessments.

For disability assessments, the choice of instrument varied depending on the condition being treated. In studies focused on low back pain, the most used tools were the Oswestry Disability Index (ODI), the Roland Morris Disability Questionnaire (RMDQ), and the Quebec Back Pain Disability Scale, with the RMDQ being the most frequently employed due to its reliability and replicability in chronic pain populations. In studies on neck pain, the Neck Disability Index (NDI) was the most widely used instrument, while the Headache Disability Index was used in one study. Additional condition-specific instruments included the Craniofacial Pain and Disability Inventory; the Disabilities of the Arm, Shoulder, and Hand Questionnaire (DASH); and the Boston Carpal Tunnel Questionnaire.

*Limitations and Strengths*

The main limitation of this study is the lack of personalization in the implementation of pain neuroscience education [45]. Variability in intervention protocols, small sample sizes, and inconsistency in follow-up conditions affect the generalizability of the results [46]. Moreover, the limited coherence among interventions may have influenced the outcomes [47]. More systematic and individualized approaches are the ones needed to address not only the physical dimensions but also the psychological and social aspects that shape the pain experience [46].

This study presents several strengths, including its adherence to PRISMA 2020 guidelines and the inclusion of randomized controlled trials, which enhance methodological rigor and internal validity. A comprehensive search across six major databases ensured broad coverage of the relevant literature, while the dual-review process minimized selection bias. The use of meta-analytic techniques with random effects models allowed for robust synthesis despite heterogeneity. Additionally, the study evaluated both physical and psychological outcomes, such as kinesiophobia and pain-related beliefs, and provided valuable insights into the use of measurement instruments for pain and disability. These strengths contribute to the reliability and relevance of the findings for clinical and research settings.

A notable strength of this review is the high methodological quality of all included trials, as indicated by PEDro scores above nine. This uniformity reduced the need for risk-based exclusions and supports the validity of the pooled estimates.

*Future research*

Future research should focus on experimental studies that assess the long-term effects of combining PNE with physical therapy. It is crucial to develop standardized treatment protocols that are applicable across different pathologies and clinical contexts. To this end, future studies should address the various stages of intervention and detail the procedures used, enabling the establishment of robust, evidence-based recommendations to enhance clinical outcomes in patients with chronic pain.

## 5. Conclusions

It can be concluded that the use of pain neuroscience education (PNE) in combination with various physical therapy modalities has a positive effect on pain management in patients with chronic pain. Moreover, the effects on disability appear to be more enduring over time compared to the effects on pain intensity.

Although no substantial differences were observed regarding the choice of pain measurement instruments, the most frequently used tools were the Visual Analog Scale (VAS) and the Numerical Pain Rating Scale (NPRS). For disability assessments, the Roland Morris Disability Questionnaire (RMDQ) was the most commonly used for lumbar conditions, while the Neck Disability Index (NDI) was the most frequent choice for cervical pathologies. In the case of other conditions, the limited number of studies prevents conclusive findings regarding the most appropriate instruments.

The effectiveness of interventions is influenced by the lack of standardization in the application of PNE, as well as by the absence of long-term follow-up in several studies. Therefore, further research is needed to distinguish the effectiveness of PNE combined with physiotherapy from physiotherapy alone. This will facilitate the development of standardized intervention protocols and improve consistency in data collection and outcome measurement across clinical contexts.

## Figures and Tables

**Table 1:** Sociodemographic characteristics of studies.

Author	Journal (Q)	Country	Age	n	Pain Type	Treatment	Pain Test	Pre-Test Pain	Post-Test Pain	Disability Test	Pre-Test Disability	Post-Test Disability
Aliyu et al., 2018 [16].	Fisioterapia (4)	Nigeria	44.26	19	Chronic low back pain	Cognitive Behavioral Therapy + Lumbar Stabilization Exercises	VAS	M: 6.21; SD: 1.40	M: 3.11; SD: 1.24	ODI	M: 43.53; SD: 11.6	M: 27.16; SD: 9.14
Aguiar et al., 2023 [17].	The Journal of Pain (1)	Brazil	38.2	74	Chronic painful temporomandibular disorders (TMD)	Pain Science Education + Manual Therapy + Therapeutic Exercise	NPRS	M: 6.2; SD: 2.1	M. 1.9; SD: 2.1	CPDI	M: 6.21; SD: 1.40	M: 6.21; SD: 1.40
Brage et al., 2015 [18].	Musculoskeletal Science and Practice (1)	Denmark	42.14	7	Neck pain	Pain Neuroscience Education + Therapeutic Exercise	NPRS	M: 4.71; SD: 1.60	M: 2.57; SD: 1.90	NDI	M: 14.29; SD: 2.81	M: 12.71; SD: 8.14
Bodes et al., 2018 [19].	Archives of Physical Medicine and Rehabilitation (1)	Spain	44.9	28	Chronic low back pain	Pain Neuroscience Education + Therapeutic Exercise	NPRS	M: 7.9; SD: 1.2	M: 5.3; SD: 1.2	RMDQ	M: 6.21; SD: 1.40	M: 6.21; SD: 1.40
Bagg et al., 2022 [20].	Journal of the American Medical Association (1)	Australia	44.7	128	Chronic low back pain	Pain Education + Gradual Sensorimotor Retraining Intervention	NPRS	M: 5.6; SD: 1.8	M: 3.1; SD: 2.4	RMDQ	M: 9.6; SD: 5.4	M: 3.6; SD: 4.6
Dilek et al., 2017 [21]	*Journal of Hand Therapy* (1)	Turkey	52.59	17	Distal radius fracture	Graded Motor Imagery + Traditional Care	VAS	6.94; SD: 1.34	M: 0.77; SD: 1.09	DASH	M: 70.65; SD: 16.8	M: 32.65; SD: 13
Galán et al., 2020 [22]	*Journal of Clinical Medicine* (1)	Spain	53.02	69	Chronic spinal pain	Pain Neuroscience Education + Therapeutic Exercise	VAS	M: 7.41; SD: 1.45	M: 2.7; SD: 1.62	RMDQ	M: 9.2; SD: 4.8	M: 3.3; SD: 3.5
Ghasemi et al., 2023 [23]	*Middle East Journal of Rehabilitation and Health Studies* (4)	Iran	46.33	15	Cervicogenic headache	Pain Neuroscience Education + Traditional Physiotherapy	VAS	M: 6.8; SD: 1.47	M: 4.07; SD: 2.09	HDI	M: 39.87; SD: 13.4	M: 22.93; SD: 12.6
Gorji et al., 2022 [24]	*International Journal of Environmental Research and Public Health* (2)	Iran	55.16	18	Chronic low back pain	Pain Neuroscience Education + Motor Control Exercises	VAS	M: 5.16; SD: 0.70	M: 2.16; SD: 0.72	RMDQ	M: 14.38; SD: 1.94	M: 6.61; SD: 0.92
Imai et al., 2021 [25]	*Journal of Occupational Health* (2)	Japan	N/E	53	Musculoskeletal pain	Pain Neuroscience Education + Therapeutic Exercise	NPRS	M: 4.6; SD: 2.6	M: 3; SD: 1.9	N/I	N/I	N/I
Matias et al., 2019 [26]	*International Journal of Therapy and Rehabilitation* (3)	Portugal	20.7	22	Neck pain	Pain Neuroscience Education + Therapeutic Exercise	VAS	M: 4; SD: 2.3	M: 3; SD: 2.3	NPDS	M: 20.4; SD: 8.1	M: 15.2; SD: 9.5
Meise et al., 2023 [27]	*Cephalalgia* (1)	Germany	45.0	47	Migraine with or without neck pain	Pain Neuroscience Education + Physiotherapy	VAS	M: 5.2; SD: 1.3	M: 4.4; SD: 1.4	NPDS	M: 13.7; SD: 4.1	M: 10.8; SD: 4
Núñez et al., 2023 [28]	*Musculoskeletal Science and Practice* (1)	Chile	45.9	12	Carpal Tunel Syndrome awaiting surgery	Pain Neuroscience Education + Therapeutic Exercise	NPRS	M: 6.8, SD: 1.9	M: 4.8; SD: 1.7	BCTQ	M: 3.4; SD: 0.7	M: 2.5; SD: 0.5
Pires et al., 2014 [29]	*Clinical Rehabilitation* (1)	Portugal	50.9	32	Chronic low back pain	Pain Neuroscience Education + Aquatic Exercise	VAS	M: 4.34; SD: 2.3	M: 2.06; SD. 1.9	QBPDS	M: 32.3; SD: 14	M: 21.2; SD: 15.8
Rabiei et al., 2021 [30]	*Pain Practice* (2)	Iran	42.46	37	Chronic low back pain	Pain Neuroscience Education + Motor Control Exercises	VAS	6.45; SD: 1.21	M: 3.79; SD: 1.02	RMDQ	M: 14.6; SD: 1.55	M: 7.34; SD: 2.17
Song et al., 2023 [31]	*Biomedicine* (1)	Korea	45.64	14	Chronic low back pain	Pain Neuroscience Education + Soft Tissue Mobilization	NPRS	M: 4.73; SD: 0.90	M: 1.78; SD: 0.50	RMDQ	M: 9.71; SD: 2.46	M: 5.07; SD: 1.38
Supe et al., 2023 [32]	*Journal of Mid-Life Health* (3)	India	58.34	35	Knee osteoarthritis	Pain Neuroscience Education + Therapeutic Exercise	NPRS	M: 6.71; SD: 1.18	M: 3.17; SD: 1.05	N/I	N/I	N/I
Tavares et al., 2023 [33]	*Brazilian Journal of Physical Therapy* (1)	Brazil	38.81	45	Chronic low back pain	Pain Neuroscience Education + Spinal Manipulation	NPRS	6.63; SD: 1.92	M: 3.40; SD: 2.24	ODI	M: 26.69; SD: 14.1	M: 13.03; SD: 11.4
Valiente-Castrillo et al., 2021 [34]	*Acupuncture in Medicine* (2)	Spain	40.35	21	Neck pain	Pain Neuroscience Education + Dry Needling	VAS	M: 5.52; SD: 1.80	M: 2.47; SD: 2.31	NPDS	M: 16.29; SD: 4.71	M: 7.57; SD: 6.19

Note: Q (SJR Quartile), Age (Mean), n (Intervention Group). Pain Tests: VAS (Visual Analog Scale), NPRS (Numerical Pain Scale). Disability Tests: ODI (Oswestry Disability Index), CPDI (Craniofacial Pain and Disability Inventory), NDI (Neck Disability Index), RMDQ (Roland Morris Disability Questionnaire), DASH (Disabilities of the Arm, Shoulder, and Hand Questionnaire), HDI (Headache Disability Index), NPDS (Neck Pain and Disability Scale), BCTQ (Boston Carpal Tunnel Questionnaire), QBPDS (Quebec Back Pain Disability Scale). Pain Tests: VAS (Visual Analog Scale), NPRS (Numerical Pain Rating Scale). Pre- and post-treatment means of pain and disability: M (Mean), SD (standard deviation), N/I (No information).

**Table 2:** Descriptive data of the quantitative variables of the study.

						Shapiro–Wilk		Percentiles	
	Mean	Median	DE	Min	Max	W	*p*	25th	75th
n	72.32	55	59.147	15	257	0.789	<0.001	36.50	86.00
Age	44.97	44.95	8.235	207.000	58.34	0.891	0.040	42.22	49.76
Pain 1	5.89	6.20	1.117	40.000	7.90	0.960	0.574	4.95	6.75
SD 1	1.60	1.47	0.502	0.7000	2.60	0.977	0.896	1.25	1.91
Pain 2	3.03	3.00	1.112	0.7700	5.30	0.982	0.967	2.32	3.59
SD 2	1.58	1.70	0.649	0.0720	2.40	0.931	0.181	1.15	2.09
Disab 1	56.67	14.38	158.607	34.000	670.65	0.319	<0.001	12.00	26.69
SD 1	6.87	4.80	5.305	0.7000	16.76	0.893	0.052	2.46	11.60
Disab 2	12.51	10.80	8.794	25.000	32.65	0.900	0.067	6.61	15.20
SD 2	6.49	6.19	4.791	0.5000	15.80	0.936	0.275	2.17	9.50

Note: SD (standard deviation); pain 1 and 2 (mean pain before and after the intervention); disability pre-test and post-test (mean pain disability before and after the intervention).

**Table 3:** Random effects model and statistics of model heterogeneity.

	Model 1	Model 2	Model 3	Model 4
Estimate	0.0588	−0.784	1.75	−0.435
se	0.0900	0.153	1.93	0.142
Z	0.654	−5.12	0.906	−3.06
*p*	0.522	<0.001	0.378	0.007
CI Lower Bound	−0.130	−1.105	−2.346	−0.736
CI Upper Bound	0.248	−0.462	5.850	−0.134
Tau	0.314	0.603	7.257	0.531
Tau^2^	0.0984 (SE = 0.051)	0.3641 (SE = 0.1441)	52.6641 (SE = 18.8317)	0.2821 (SE = 0.1231)
I^2^	69.28%	88.58%	99.91%	85.69%
H^2^	3.255	8.755	1.095.824	6.989
df	18.000	18.000	16.000	16.000
Q	56.745	144.398	230.573	99.719
*p*	<0.001	<0.001	<0.001	<0.001

Note: Tau^2^ estimator: restricted maximum plausibility.

**Table 4:** Evaluation of the publication bias of the models.

	Model 1		Model 2		Model 3		Model 4	
Test Name	Value	*p*	Value	*p*	Value	*p*	Value	*p*
Fail-Safe N	0.000	0.140	1.356.000	<0.001	15.000	0.013	419.000	<0.001
Correlation	0.076	0.679	−0.310	0.068	0.235	0.205	0.029	0.903
Egger’s Regression	0.127	0.901	−1.641	0.119	8.895	<0.001	0.042	0.967

Note: Fail-safe N computation using the Rosenthal approach; Begg and Mazumdar Rank Correlation.

## Data Availability

No new data were generated or analyzed in this study. Therefore, data sharing is not applicable.

## Supplementary Materials

The following supporting information can be downloaded at https://www.mdpi.com/article/10.3390/brainsci15060658/s1, Supplementary S1: Search phrases; Supplementary S2: Pedro scale; Supplementary S3: GRADE assessment; Supplementary S4: Characteristics of the pain education sessions; Supplementary S5: Characteristics of the treatments; Supplementary S6: Prisma (Check List) [10].

## Abbreviations

The following abbreviations are used in this manuscript:

PNE	Pain Neuroscience Education
VAS	Visual Analog Scale
NAS	Numerical Analog Scale
NPRS	Numerical Pain Rating Scale
RMQ	Roland Morris Disability Questionnaire
ODI	Oswestry Disability Index
NDI	Neck Disability Index
DASH	Disabilities of the Arm, Shoulder, and Hand Questionnaire
HAI	Headache Disability Index
CPTQ	Boston Carpal Tunnel Questionnaire
CBP	Chronic Back Pain
CBT	Cognitive Behavioral Therapy
LSE	Lumbar Stabilization Exercise
RESOLVE	Gradual Sensorimotor Retraining Intervention
GMI	Graded Motor Imagery
MCEs	Motor Control Exercises
PRISMA	Preferred Reporting Items for Systematic Reviews and Meta-Analyses
PICOS	Population, Intervention, Comparison, Outcomes, Study Design
WHO	World Health Organization
IASP	International Association for the Study of Pain
SJR	Scimago Journal Rank
SD	Standard Deviation
CI	Confidence Interval
df	Degrees of Freedom
