# Supplementary material for: Effectiveness of Pain Neuroscience Education in Physical Therapy: A Systematic Review and Meta-Analysis
Source: Brain Sci. 2025 Jun 18;15(6):658. doi: 10.3390/brainsci15060658 (PMC12191368; doi:10.3390/brainsci15060658)
Supplement: Supplementary file 1 [file brainsci-15-00658-s001.zip › brainsci-3644710-supplementary.pdf]

Effectiveness of Pain Neuroscience Education in Physical Therapy: A Systematic Review and Meta-Analysis

Andrea Sánchez-Robalino [1]

Hugo Sinchi-Sinchi [2]

Andrés Ramírez [3 *]

1 Department of Physiotherapy, Pontificia Universidad Católica del Ecuador Sede Esmeraldas, 080101Esmeraldas, Ecuador.

2 Department of Psychology, Pontificia Universidad Católica del Ecuador Sede Esmeraldas, 080101Esmeraldas, Ecuador.

3 Department of Clinical Psychology, Universidad Politécnica Salesiana, 010107 Cuenca, Ecuador

*Correspondence: Andrés Ramírez. aramirezc1@ups.edu.ec, arc04878@gmail.com. Panamericana Norte, Cuenca- Ecuador

Appendix

Supplementary S1. Search phrases

Supplementary S2. Pedro Scale

Supplementary S3. GRADE Assessment

Supplementary S4. Characteristics of the Pain Education sessions

Supplementary S5. Characteristics of the treatments

Supplementary S6. Prisma (Check List)

Supplementary S1. Search phrases

Data Bases	Search Legend	n
Scopus	TITLE-ABS-KEY ("Chronic Pain" OR "Musculoskeletal Pain" OR "Chronic Disease") AND TITLE-ABS-KEY ("Physical Therapy Modalities" OR "Pain Management" OR "Rehabilitation") AND TITLE-ABS-KEY ("Education" OR "Neurosciences" OR "Neurobiology") AND (LIMIT-TO (DOCTYPE , "ar")) AND (LIMIT-TO (EXACTKEYWORD , "Randomized Controlled Trial") OR LIMIT-TO (EXACTKEYWORD , "Randomized Controlled Trial (topic)") OR LIMIT-TO (EXACTKEYWORD , "Controlled Clinical Trial"))	442
Science Direct	("Chronic Pain" OR "Musculoskeletal Pain" OR "Chronic Disease") AND ("Physical Therapy Modalities" OR "Pain Management" OR "Rehabilitation") AND ("Education" OR "Neurosciences" OR "Neurobiology")	649
Web Of Science	("Chronic Pain" OR "Musculoskeletal Pain" OR "Chronic Disease") AND ("Physical Therapy Modalities" OR "Pain Management" OR "Rehabilitation") AND ("Education" OR "Neurosciences" OR "Neurobiology")<br><br>Document Types: Article AND Document Types: Clinical Trial AND MeSH Headings: Adult	334
PsyINFO	("Chronic Pain" OR "Musculoskeletal Pain" OR "Chronic Disease") AND ("Physical Therapy Modalities" OR "Pain Management" OR "Rehabilitation") AND ("Education" OR "Neurosciences" OR "Neurobiology")<br><br>Source Types: Academic Journals AND Methodology: quantitative study<br><br>Subject; chronic pain AND pain management AND rehabilitation AND education AND clinical trials	163
Cochrane	("Chronic Pain" OR "Musculoskeletal Pain" OR "Chronic Disease") AND ("Physical Therapy Modalities" OR "Pain Management" OR "Rehabilitation") AND ("Education" OR "Neurosciences" OR "Neurobiology") in Keyword	306
Pubmed	Search: ("Chronic Pain" OR "Musculoskeletal Pain" OR "Chronic Disease") AND ("Physical Therapy Modalities" OR "Pain Management" OR "Rehabilitation") AND ("Education" OR "Neurosciences" OR "Neurobiology") Filters: Randomized Controlled Trial, Adult: 19+ years Sort by: Publication Date	534
Total		2428

Supplementary S2. Pedro Scale

Pedro Scale																							
**Study**	1	Where:	2	Where:	3	Where:	4	Where:	5	Where:	6	Where:	7	Where:	8	Where:	9	Where:	10	Where:	11	Where:	TOTAL
**1**	1	Participants	1	Randomization	1	Randomization	1	Participants	0		1	Outcome Measures	1	Outcome Measures	1	Statistical analyses	1	Procedure	1	Statistical analyses	1	Results	10
**2**	1	Participants	1	Design	1	Procedures, Randomization and Allocation	1	Sample Size	0		0		1	Procedures, Randomization and Allocation	1	Procedures, Randomization and Allocation	1	Outcomes	1	Outcomes	1	Outcomes	9
**3**	1	Procedu	1	Procedu	1	Procedu	1	Statistical analysis	0		0		1	Procedures	1	Outcomes	1	Outcomes	1	Statistical analysis	1	Results	9
**4**	1	Methods	1	Methods	1	Randomization and blinding	1	Randomization and blinding	0		0		0		1	Results	1	Results	1	Results	1	Results	8
**5**	1	Methods	1	Randomization and Masking	1	Randomization and Masking	1	Study Design and Participants	1		0		1	Study Design and Participants	1	Outcome Measurements	1	Outcome Measurements	1	Outcome Measurements	1	Outcome Measurements	10
**6**	1	Methods	1	Methods	1	Methods	1	Methods	1	Methods	0		0		1	Results	1	Results	1	Resulys	1	Results	9
**7**	1	Experimental section	1	Experimental section	1	Experimental section	1	Experimental section	0		0		1	Experimental section	1	Outcome variables	1	Outcome variables	1	Outcome variables	1	Statistical analysis	9
**8**	1	Methods	1	Methods	1	Methods	1	Methods	0		0		0		1	Resu	1	Resu	1	Resu	1	Resu	8

**9**	1	Materials and methods	1	Materials and methods	1	Materials and methods	1	Materials and methods	0		0		0		1	Results	1	Results	1	Results	1	Results	8
**10**	1	Participants	1	Randomization and blinding	1	Randomization and blinding	1	Materials and methods	0		0		1	Randomization and blinding	1	Outcome measurements	1	Outcome measurements	1	Results	1	Results	9
**11**	1	Methods	1	Methods	1	Methods	1	Methods	0		0		1	Methods	1	Results	1	Results	1	Results	1	Treatment effects/outcomes	9
**12**	1	Methods	1	Methods	1	Methods	1	Methods	0		0		0		1	Methods	1	Methods	1	Results	1	Results	8
**13**	1	Methods	1	Methods	1	Methods	1	Methods	0		0		0		1	Results	1	Results	1	Results	1	Results	8
**14**	1	Methods	1	Methods	1	Methods	1	Methods	0		0		1	Methods	1	Results	1	Results	1	Results	1	Results	9
**15**	1	Material and methods	1	Material and methods	1	Material and methods	1	Sample size calculation and statistical analysis	0		0		1	Interventions	1	Outcome measurements	1	Outcome measurements	1	Results	1	Results	9
**16**	1	Material and methods	1	Material and methods	1	Material and methods	1	Material and methods	0		0		1	Material and methods	1	Results	1	Results	1	Results	1	Results	9
**17**	1	Materials and methods	1	Materials and methods	1	Materials and methods	1	Materials and methods	1	Blinding	0		1	Blinding	1	Results	1	Results	1	Results	1	Results	10
**18**	1	Methods	1	Randomization, allocation, and blinding	1	Randomization, allocation, and blinding	1	Statistical analysis	0		1	Methods	1	Results	1	Results	1	Results	1	Results	1	Results	10

**19**	1	Methods	1	Methods	1	Methods	1	Methods	0		0		1	Methods	1	Methods	1	Results	1	Results	1	Results	9

Note: 1) Eligibility criteria were specified; 2) Subjects were randomly allocated to groups (in a crossover study, subjects were randomly allocated an order in which treatments were received); 3) Allocation was concealed; 4) The groups were similar at baseline regarding the most important prognostic indicators; 5) There was blinding of all subjects; 6) There was blinding of all therapists who administered the therapy; 7) There was blinding of all assessors who measured at least one key outcome; 8) Measures of at least one key outcome were obtained from more than 85% of the subjects initially allocated to groups; 9) All subjects for whom outcome measures were available received the treatment or control condition as allocated or, where this was not the case, data for at least one key outcome was analysed by "intention to treat"; 10) The results of between-group statistical comparisons are reported for at least one key outcome; 11) The study provides both point measures and measures of variability for at least one key outcome.

Supplementary S3. GRADE Assessment

Autor(es): Andrea Sanchez, Hugo Sinchi, Andrés Ramírez
Pregunta: Pain Neuroscience Education + physiotherapy comparado con Physiotherapy alone para adults with chronic pain
Configuración: clinical rehabilitation settings or outpatient clinics
Bilbiografía:

Evaluación de certeza							№ de pacientes		Efecto		Certeza	Importancia
№ de estudios	Diseño de estudio	Riesgo de sesgo	Inconsistencia	Evidencia indirecta	Imprecisión	Otras consideraciones	Pain Neuroscience Education + physiotherapy	Physiotherapy alone	Relativo (95% CI)	Absoluto (95% CI)		
**Pain reduction (seguimiento: 6; evaluado con : NPRS or VAS (standardized to 0-10 scale); Escala de: 0 a 10)**												
19	ensayos aleatorios	no es serio	serio	no es serio	no es serio	ninguno	346	347	-	MD **0.784 más alto.** (1.105 más alto. a 0.462 más alto.)	⨁⨁⨁◯ Moderado	NO IMPORTANTE

CI: Intervalo de confianza ; **MD:** Diferencia media

Supplementary S4. Characteristics of the Pain Education sessions

Papers	Pain Neuroscience Education
Aliyu et al. 2018	Cognitive Behavioral Therapy, 12 sessions
Aguiar et al. 2023	Pain Science Education, 2 sessions
Brage et al. 2015	Pain Neuroscience Education, 4 sessions
Bodes Pardo et al. 2018.	Pain Neuroscience Education, 2 sessions
Bagg et al. 2022	Pain Education, 1 session
Dilek et al. 2018	Graded Motor Imagery, does not report number of sessions
Galan-Martin et al. 2020	Pain Neuroscience Education, 6 sessions
Ghasemi et al. 2023	Pain Neuroscience Education, 4 sessions
Gorji et al. 2022	Pain Neuroscience Education, 3 sessions
Imai et al. 2021	Pain Neuroscience Education, 1 session
Matias et al. 2019	Pain Neuroscience Education, 4 sessions
Meise et al. 2023	Pain Neuroscience Education, 6 sessions
Núñez-Cortés et al. 2023	Pain Neuroscience Education, 3 sessions
Pires et al. 2015	Pain Neuroscience Education, 2 sessions
Rabiei et al. 2021	Pain Neuroscience Education, 3 sessions
Song et al. 2023	Pain Neuroscience Education, 2 sessions
Supe et al. 2023	Pain Neuroscience Education, 2 sessions
Tavares et al. 2023	Pain Neuroscience Education, 2 sessions
Valiente-Castrillo et al. 2021	Pain Neuroscience Education, 3 sessions

Note: Heterogeneity in the number of sessions, it should be noted that each author delivers the information in a variable manner in terms of time, content and mode (personalized or group).

Supplementary S5. Characteristics of the treatments

	Intervention	Control
Aliyu et al. 2018	Cognitive Behavioral Therapy+ Lumbar Stabilization Exercises	Lumbar Stabilization Exercises
Aguiar et al. 2023	Pain Science Education + Manual Therapy + Therapeutic Exercise	Manual Therapy + Therapeutic Exercise
Brage et al. 2015	Pain Neuroscience Education + Therapeutic Exercise	Pain Neuroscience Education
Bodes Pardo et al. 2018.	Pain Neuroscience Education + Therapeutic Exercise	Therapeutic Exercise
Bagg et al. 2022	Pain Education + Gradual Sensorimotor Retraining Intervention	Pain discussion without education+ Simulated physical agents+ Simulated cranial electrical stimulation device.
Dilek et al. 2018	Graded Motor Imagery + Traditional Care	Traditional physiotherapy
Galan-Martin et al. 2020	Pain Neuroscience Education + Therapeutic Exercise	Traditional physiotherapy
Ghasemi et al. 2023	Pain Neuroscience Education + Traditional physiotherapy	Traditional physiotherapy
Gorji et al. 2022	Pain Neuroscience Education + Motor Control Exercises	Core stabilization training
Imai et al. 2021	Pain Neuroscience Education + Therapeutic Exercise	
Matias et al. 2019	Pain Neuroscience Education + Therapeutic Exercise	Therapeutic Exercise
Meise et al. 2023	Pain Neuroscience Education + Physiotherapy	Traditional physiotherapy
Núñez-Cortés et al. 2023	Pain Neuroscience Education + Therapeutic Exercise	Therapeutic Exercise
Pires et al. 2015	Pain Neuroscience Education + Aquatic Exercise	Aquatic Exercise
Rabiei et al. 2021	Pain Neuroscience Education + Motor Control Exercises	Motor Control Exercises
Song et al. 2023	Pain Neuroscience Education + Soft Tissue Mobilization	Soft Tissue Mobilization
Supe et al. 2023	Pain Neuroscience Education + Therapeutic Exercise	Therapeutic Exercise
Tavares et al. 2023	Pain Neuroscience Education + Spinal Manipulation	Spinal Manipulation
Valiente-Castrillo et al. 2021	Pain Neuroscience Education + Dry Needling	Dry Needling

Note: The treatments used are described according to the intervention and control groups.

Supplementary S6. Prisma (Check List)

Section and Topic	Item #	Checklist item	Location where item is reported
**TITLE**			
Title	1	Identify the report as a systematic review.	1
**ABSTRACT**			
Abstract	2	See the PRISMA 2020 for Abstracts checklist.	1
**INTRODUCTION**			
Rationale	3	Describe the rationale for the review in the context of existing knowledge.	2
Objectives	4	Provide an explicit statement of the objective(s) or question(s) the review addresses.	2
**METHODS**			
Eligibility criteria	5	Specify the inclusion and exclusion criteria for the review and how studies were grouped for the syntheses.	2
Information sources	6	Specify all databases, registers, websites, organisations, reference lists and other sources searched or consulted to identify studies. Specify the date when each source was last searched or consulted.	3
Search strategy	7	Present the full search strategies for all databases, registers and websites, including any filters and limits used.	3
Selection process	8	Specify the methods used to decide whether a study met the inclusion criteria of the review, including how many reviewers screened each record and each report retrieved, whether they worked independently, and if applicable, details of automation tools used in the process.	3
Data collection process	9	Specify the methods used to collect data from reports, including how many reviewers collected data from each report, whether they worked independently, any processes for obtaining or confirming data from study investigators, and if applicable, details of automation tools used in the	3

Section and Topic	Item #	Checklist item	Location where item is reported
		process.	
Data items	10a	List and define all outcomes for which data were sought. Specify whether all results that were compatible with each outcome domain in each study were sought (e.g. for all measures, time points, analyses), and if not, the methods used to decide which results to collect.	4
	10b	List and define all other variables for which data were sought (e.g. participant and intervention characteristics, funding sources). Describe any assumptions made about any missing or unclear information.	4
Study risk of bias assessment	11	Specify the methods used to assess risk of bias in the included studies, including details of the tool(s) used, how many reviewers assessed each study and whether they worked independently, and if applicable, details of automation tools used in the process.	5
Effect measures	12	Specify for each outcome the effect measure(s) (e.g. risk ratio, mean difference) used in the synthesis or presentation of results.	
Synthesis methods	13a	Describe the processes used to decide which studies were eligible for each synthesis (e.g. tabulating the study intervention characteristics and comparing against the planned groups for each synthesis (item #5)).	
	13b	Describe any methods required to prepare the data for presentation or synthesis, such as handling of missing summary statistics, or data conversions.	
	13c	Describe any methods used to tabulate or visually display results of individual studies and syntheses.	
	13d	Describe any methods used to synthesize results and provide a rationale for the choice(s). If meta-analysis was performed, describe the model(s), method(s) to identify the presence and extent of statistical heterogeneity, and software package(s) used.	
	13e	Describe any methods used to explore possible causes of heterogeneity among study results (e.g. subgroup analysis, meta-regression).	
	13f	Describe any sensitivity analyses conducted to assess robustness of the synthesized results.	
Reporting bias assessment	14	Describe any methods used to assess risk of bias due to missing results in a synthesis (arising from reporting biases).	5
Certainty assessment	15	Describe any methods used to assess certainty (or confidence) in the body of evidence for an outcome.	5

Section and Topic	Item #	Checklist item	Location where item is reported
**RESULTS**			
Study selection	16a	Describe the results of the search and selection process, from the number of records identified in the search to the number of studies included in the review, ideally using a flow diagram.	4
	16b	Cite studies that might appear to meet the inclusion criteria, but which were excluded, and explain why they were excluded.	6
Study characteristics	17	Cite each included study and present its characteristics.	5
Risk of bias in studies	18	Present assessments of risk of bias for each included study.	6-8
Results of individual studies	19	For all outcomes, present, for each study: (a) summary statistics for each group (where appropriate) and (b) an effect estimate and its precision (e.g. confidence/credible interval), ideally using structured tables or plots.	6-16
Results of syntheses	20a	For each synthesis, briefly summarise the characteristics and risk of bias among contributing studies.	5-8
	20b	Present results of all statistical syntheses conducted. If meta-analysis was done, present for each the summary estimate and its precision (e.g. confidence/credible interval) and measures of statistical heterogeneity. If comparing groups, describe the direction of the effect.	5-8
	20c	Present results of all investigations of possible causes of heterogeneity among study results.	5-8
	20d	Present results of all sensitivity analyses conducted to assess the robustness of the synthesized results.	5-8
Reporting biases	21	Present assessments of risk of bias due to missing results (arising from reporting biases) for each synthesis assessed.	
Certainty of evidence	22	Present assessments of certainty (or confidence) in the body of evidence for each outcome assessed.	5-8
**DISCUSSION**			
Discussion	23a	Provide a general interpretation of the results in the context of other evidence.	8

Section and Topic	Item #	Checklist item	Location where item is reported
	23b	Discuss any limitations of the evidence included in the review.	8
	23c	Discuss any limitations of the review processes used.	9
	23d	Discuss implications of the results for practice, policy, and future research.	9
**OTHER INFORMATION**			
Registration and protocol	24a	Provide registration information for the review, including register name and registration number, or state that the review was not registered.	
	24b	Indicate where the review protocol can be accessed, or state that a protocol was not prepared.	
	24c	Describe and explain any amendments to information provided at registration or in the protocol.	
Support	25	Describe sources of financial or non-financial support for the review, and the role of the funders or sponsors in the review.	19
Competing interests	26	Declare any competing interests of review authors.	18
Availability of data, code and other materials	27	Report which of the following are publicly available and where they can be found: template data collection forms; data extracted from included studies; data used for all analyses; analytic code; any other materials used in the review.	18

From: Page MJ, McKenzie JE, Bossuyt PM, Boutron I, Hoffmann TC, Mulrow CD, et al. The PRISMA 2020 statement: an updated guideline for reporting systematic reviews. BMJ 2021;372:n71. doi: 10.1136/bmj.n71. This work is licensed under CC BY 4.0. To view a copy of this license, visit https://creativecommons.org/licenses/by/4.0/
